# Vaccine hesitancy and equity: lessons learned from the past and how they affect the COVID-19 countermeasure in Indonesia

**DOI:** 10.1186/s12992-023-00987-w

**Published:** 2024-02-06

**Authors:** Rano K. Sinuraya, Rina F. Nuwarda, Maarten J. Postma, Auliya A. Suwantika

**Affiliations:** 1 Unit of Global Health, Department of Health Sciences, University of Groningen, University Medical Center Groningen, Groningen, the Netherlands; 2 Department of Pharmacology and Clinical Pharmacy, Faculty of Pharmacy, Universitas Padjadjaran, Jalan Ir. Soekarno KM 21, Jatinangor, Sumedang, West Java 45363 Indonesia; 3 Center of Excellence for Pharmaceutical Care Innovation, Universitas Padjadjaran, Sumedang, West Java Indonesia; 4 Sydney Pharmacy School, Faculty of Medicine and Health, The University of Sydney, Sydney, Australia; 5 Department of Pharmaceutical Analysis and Medicinal Chemistry, Faculty of Pharmacy, Universitas Padjadjaran, Sumedang, West Java Indonesia; 6 Department of Economics, Econometrics & Finance, Faculty of Economics & Business, University of Groningen, Groningen, the Netherlands

**Keywords:** Immunization, Vaccine-preventable diseases, National immunization program, COVID-19, Vaccine hesitancy

## Abstract

**Introduction:**

Indonesia has made progress in increasing vaccine coverage, but equitable access remains challenging, especially in remote areas. Despite including vaccines in the National Immunization Program (NIP), coverage has not met WHO and UNICEF targets, with childhood immunization decreasing during the COVID-19 pandemic. COVID-19 vaccination has also experienced hesitancy, slowing efforts to end the pandemic.

**Scope:**

This article addresses the issue of vaccine hesitancy and its impact on vaccination initiatives amidst the COVID-19 pandemic. This article utilizes the vaccine hesitancy framework to analyze previous outbreaks of vaccine-preventable diseases and their underlying causes, ultimately providing recommendations for addressing the current situation. The analysis considers the differences between the pre-pandemic circumstances and the present and considers the implementation of basic and advanced strategies.

**Key findings and conclusion:**

Vaccine hesitancy is a significant challenge in the COVID-19 pandemic, and public health campaigns and community engagement efforts are needed to promote vaccine acceptance and uptake. Efforts to address vaccine hesitancy promote trust in healthcare systems and increase the likelihood of individuals seeking preventive health services. Vaccine hesitancy requires a comprehensive, culturally sensitive approach that considers local contexts and realities. Strategies should be tailored to specific cultural and societal contexts and monitored and evaluated.

## Highlights

- Vaccine hesitancy is a complex issue influenced by various factors, including a lack of trust in vaccines, misinformation, cultural beliefs, and past negative vaccine experiences.
- The hesitancy to get vaccinated could lead to low vaccination coverage and slow eradication of vaccine-preventable diseases.
- It is crucial to understand the specific drivers of each country and population to overcome vaccine hesitancy.
- Collaborating with community or religious leaders, healthcare providers, and influencers could increase people’s awareness and gain trust, which can help reduce vaccine hesitancy.

The issue of vaccine hesitancy and vaccine inequity are closely related, with the potential to significantly influence each other.

## Introduction

A vaccination program is a cost-effective health program that can boost the immune system, thereby reducing infectious disease severity or mortality rate [1]. The SAGE Working Group defined vaccine hesitancy as follows “vaccine hesitancy refers to the delay in acceptance or refusal of vaccination despite the availability of vaccination services. Vaccine hesitancy is complex and context-specific, varying across time, place, and vaccines. It is influenced by factors such as complacency, convenience and confidence” [2]. Vaccination hesitancy is a significant threat to public health, as it can lead to disease outbreaks and even the reappearance of diseases considered to have been eradicated [3, 4]. The term “vaccine hesitancy” is not applicable in cases where low vaccine uptake is caused by factors such as unavailability of vaccines, lack of access to vaccination services, inconvenient travel or long distances to clinics, and inadequate communication [5].

Despite previous incidents of vaccine refusal contributing to measles and polio outbreaks in Indonesia [6], the country has made substantial progress in improving vaccine coverage and reducing the prevalence of vaccine-preventable diseases in recent years. The national average immunization coverage has now reached 80%, resulting in a notable decline in reported cases of diseases like pertussis, tetanus, measles, and rubella in 2019 compared to previous years [7].

The Indonesian government has introduced various programs and initiatives aimed at increasing vaccine coverage and eradicating vaccine-preventable diseases, including the inclusion of some vaccines in the National Immunization Program (NIP) [7, 8]. Despite incorporating multiple vaccines into the NIP, immunization coverage has yet to meet the targets established by the World Health Organization (WHO) and the United Nations Children’s Fund (UNICEF) [7, 9–12].

**Fig. 1:**
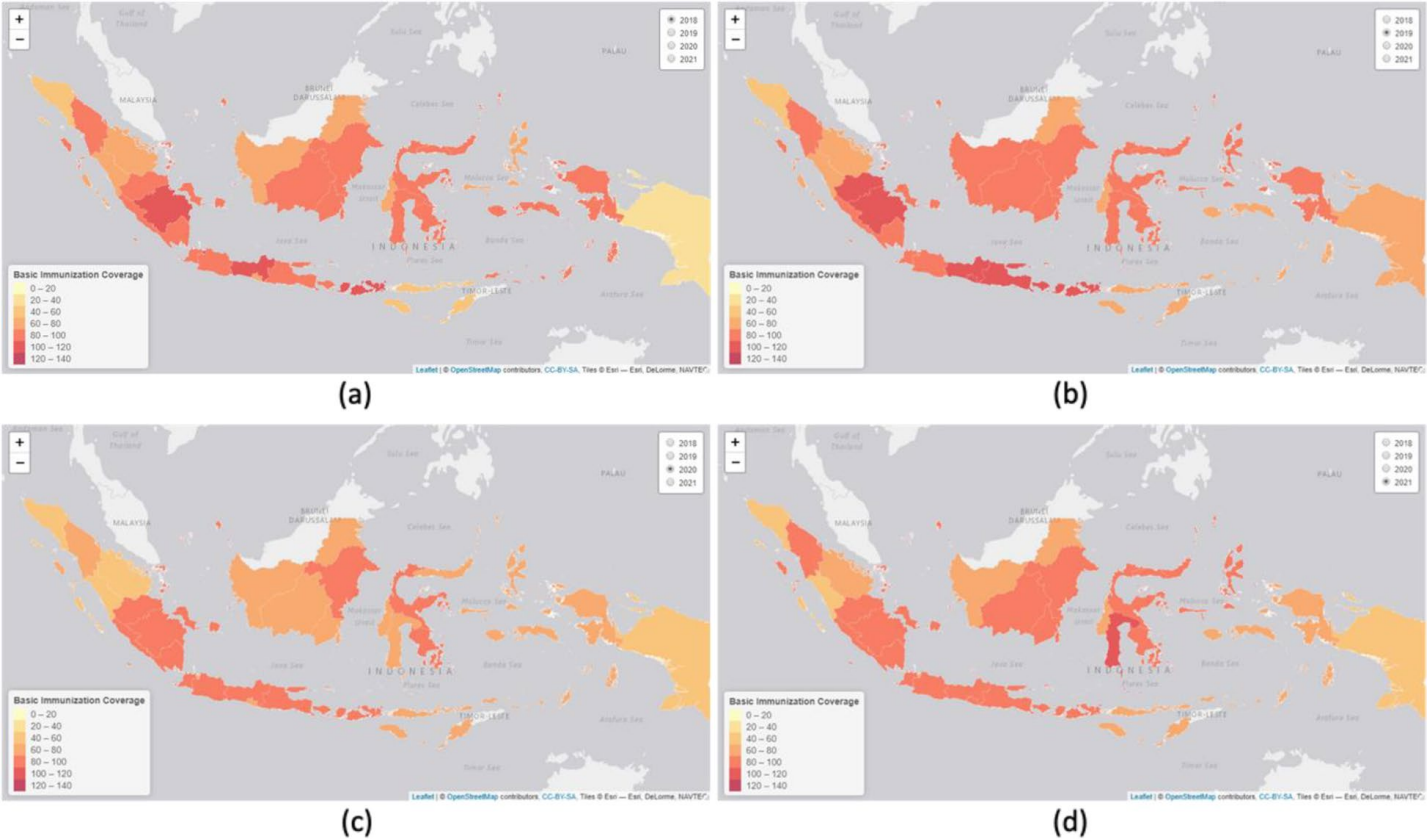
The percentage of basic immunization coverage in Indonesia (2018–2021). The information is obtained from Indonesia’s health profile 2018–2021, which is then depicted as a map showing the distribution of vaccinations across the different provinces in the country [9–12]

In March 2020, Indonesia reported its first case of COVID-19, and the country introduced its vaccination program in early 2021 [13, 14]. The global emergence of COVID-19 as a pandemic profoundly impacted childhood vaccination rates in nearly all provinces of Indonesia, as demonstrated in Fig. 1. The data reveals a notable decline in vaccination coverage in 2020 compared to previous years. However, there was a positive shift in 2021, aligning with the introduction of COVID-19 vaccines.

Similar to regular immunization, the COVID-19 vaccination program also experienced hesitation, which has slowed efforts to end the pandemic, increased the risk of infection, and negatively impacted the global economy [15]. Coverage of the first dose of COVID-19 vaccination is currently less than 90% worldwide. Indonesia, the fourth most populated country in the world, has a COVID-19 vaccination target of 234,666,020 individuals, or at least 70% of the total population [16]. The initial phase of COVID-19 vaccination in Indonesia involves the administration of Sinovac vaccine in two doses, followed by third and fourth doses as booster shots using Astra Zeneca, Pfizer-BioNTech, Moderna, or Sinopharm products [17].

In this article, we are discussing and reflecting on how the hesitancy impacts the COVID-19 vaccination program and what lessons have been learned. These lessons can help us understand how vaccine hesitancy affects the COVID-19 response in Indonesia. Although our focus is on Indonesia, many of the issues discussed below also have essential implications on a global scale, especially for low- and middle-income countries.

## Vaccine hesitancy framework

A substantial amount of study has been undertaken on vaccine hesitancy and the various elements influencing an individual’s decision to accept or not accept a vaccine. SAGE developed three categories based on experience in various countries and comprehensive literature reviews to analyze these issues, referred to as the 3Cs model: complacency (not considering diseases as high-risk and vaccination as crucial), convenience (practical obstacles), and confidence (a lack of trust in vaccine safety and effectiveness) [2, 5]. This model was later revised in 2018, emphasizing the importance of more than just the concept of confidence, and emerged as the 5Cs model (Table 1): confidence, complacency, constraints (modification of the term convenience to now include both structural and psychological barriers), calculation (individuals’ engagement in extensive information searching), and collective responsibility (communal orientation to protect others) [18, 19].

**Table 1:** Vaccine hesitancy determinants and strategies to improve it

Determinant	Point of intervention	Strategy
Confidence	Trust in vaccine safety and efficacy, the system, policy makers	Healthcare providers and public health officials can communicate transparently about the vaccine development and approval process, provide accurate information about vaccine safety and efficacy, and highlight the benefits of vaccination.
Complacency	Knowledge and general awareness	Public health officials can educate the public about the continued importance of vaccination, highlight the risks of VPDs outbreaks, and emphasize the role of vaccination in protecting community health.
Constraint	Structural and psychological barriers	Public health officials can work to make vaccines more affordable and accessible, provide mobile vaccination clinics in underserved areas, and implement policies that ensure equitable distribution of vaccines.
Risk Calculation	Willingness to protect others by one’s own vaccination	Healthcare providers can provide accurate information about vaccine safety and efficacy, share scientific studies that disprove the link between vaccines and autism, and highlight the risks of not vaccinating.
Collective Responsibility	Engagement in extensive information searching	Public health officials can emphasize the role of vaccination in protecting community health, highlight the importance of herd immunity, and address vaccine allocation and equity.

In 2016, Thomson et al. introduced a different taxonomy to explain vaccine uptake determining factors, known as the 5As. They identified five categories regarded as access (the ability of an individual to reach or be reached by vaccination), affordability (the capacity of an individual to afford vaccines either financially or non-financially), awareness (personal knowledge about the importance of vaccination as well as its objectives and risks), acceptance (the degree to accept or refuse vaccination), and activation (motivation/encouragement to receive vaccination) [20].

In the Indonesian context, there have been no studies specifically dedicated to assessing or exploring hesitancy regarding routine vaccination using the 5Cs approach. However, a recent study conducted by Sujarwoto et al. [21], which investigated COVID-19 vaccine hesitancy in a district in Indonesia, revealed that respondents held low levels of confidence and complacency beliefs about the vaccine. Furthermore, the study identified more general sources of mistrust within the community, particularly concerning health providers and vaccine developers. However, these factors may vary depending on individual, cultural, and societal contexts. By comprehending these elements, healthcare providers and public health officials can formulate precise strategies to tackle vaccine hesitancy and enhance vaccine acceptance and utilization, as presented in Table 1.

### Vaccine Hesitancy’s drivers

Misinformation and conspiracy theories are widely recognized as critical drivers of vaccine hesitancy. False information about the safety and efficacy of vaccines can spread quickly and easily through social media and other channels, which leading to fear and skepticism about vaccination [22, 23]. One prominent example of vaccine misinformation is the claim that the measles, mumps, and rubella (MMR) vaccine causes autism. As a result, some parents have refused to have their children vaccinated, which in the long term, could lead to outbreaks of measles in specific populations [24, 25].

During the COVID-19 pandemic in Indonesia, misinformation and hoaxes have contributed to vaccine hesitancy among parents and caregivers, especially concerning vaccines that require multiple injections as part of routine immunization [26, 27]. The proportion of children who received their primary measles and rubella immunizations experienced a decline from 95% in 2019 to 87% in 2021. Moreover, there has been a substantial increase in the percentage of children who were not administered the diphtheria, pertussis, and tetanus (DPT) immunizations, rising from 10% in 2019 to 26% in 2021 [27]. This situation poses a significant risk to children, as it increases their susceptibility to a range of preventable diseases.

Beside misinformation and conspiration theories, lack of trust in government and healthcare institutions could impact the vaccine hesitancy. For instance, the case of Tuskegee Syphilis Study, which was conducted by unethically on African American men, has resulted in Black communities [28] enduring mistrust of government and healthcare institutions. Similarly, in Indonesia, the lack of trust in the government has been triggered by various factors, including past conflicts in certain provinces [29] and the government’s response to the COVID-19 situation [30]. This lack of trust is exacerbated by existing disparities in healthcare access and delivery, which could lead people to be hesitant about getting the vaccine due to concerns about unequal distribution and difficulty of access [31, 32].

Next, vaccine safety and adverse effects is commonly stimulating vaccine hesitancy [33]. People may be unwilling to get immunized out because they are worried about adverse reactions, especially if they have a history of allergies or prior medical disorders [19, 33]. In the past, there have been questions about the safety of the HPV vaccine due to claims made by certain people that it can result in chronic discomfort, seizures, and even death [34]. The vaccine is safe and effective, but scientific evidence has shown that these allegations are mainly baseless [34, 35].

Similarly, concerns about the safety of the COVID-19 vaccine have been expressed, particularly in light of its rapid development and emergency use authorization [36]. Clinical studies and real-world data have consistently shown that these vaccines are highly effective with minimal risk of severe side effects [37]. However, a national survey on COVID-19 vaccine acceptance conducted by the Ministry of Health of Indonesia, which included 112,888 participants, revealed some concerning results. It showed that 64.8% were willing to take the vaccine, 7.6% were unwilling to take it, and 26.6% were unsure about whether to get vaccinated. Furthermore, participants in the survey expressed various concerns about COVID-19 vaccines. Specifically, 30% were uncertain about the vaccine’s safety, 22% had reservations about its effectiveness, 12% expressed fears of potential side effects, 8% cited religious or belief-related reasons, and 15% cited other factors [38].

In the social context, previous studies showed that cultural and religious beliefs may also play a role in vaccine hesitancy [39]. Some individuals may be hesitant to get vaccinated due to religious or cultural beliefs that conflict with vaccination, such as the belief that illness is God’s punishment or that alternative remedies are more effective than modern medicine [40].

Concerns about the use of fetal cells in vaccine development and the belief that illnesses are divine punishment have contributed to vaccine reluctance in some Orthodox Jewish communities, for instance [41, 42]. Correspondingly, in Indonesia, a country where approximately 87% of the population is Muslim, concerns have arisen over the use of non-halal components obtained from pork in vaccine formulations. These concerns have the potential to increase vaccine hesitancy in the country [43].

In addition, vaccine hesitancy may be influenced by socioeconomic variables such as low income, educational attainment, and limited healthcare accessibility [44]. Individuals residing in financially disadvantaged conditions may encounter obstacles in accessing vaccinations, such as financial constraints or scheduling conflicts that prevent them from receiving the vaccine promptly, or they may opt not to receive it [44, 45]. Individuals with lower education levels may have a restricted understanding of vaccines and their advantages, rendering them more vulnerable to misinformation [46]. In addition, inadequate healthcare accessibility may impede individuals from obtaining vaccinations on time, while restricted access to precise health information may result in misconceptions or skepticism regarding vaccines [47].

Behavioural scientists have investigated how heuristics, including vaccination, might influence judgement and decision making. Heuristics, a mental shortcut that enables people to solve problems quickly and make intuitive decisions, can be helpful when initiated by the correct variables [48, 49]. However, the influence of wrong circumstances such as misinformation and disinformation, and anti-vaccine movement, can lead to systemic errors or cognitive biases. For example, omission bias occurs when people tend to view harms from the act commission (actions) as more excellent than harms from omission (inactions); confirmation bias refers to the finding that strong initial beliefs are resistant to change because they influence how subsequent information is interpreted; and the “Dunning Krueger effect”, in which people who lack expertise fail to accurately assess their knowledge in comparison to experts on the subject [49].

Notwithstanding the unwillingness of specific individuals to receive vaccines, it is important to acknowledge the existence and impact of the anti-vaccine movement. They engage in campaigns against vaccines, frequently disseminating inaccurate information and instilling apprehension regarding their safety and efficacy [50]. The current campaign has the potential to generate vaccine hesitancy among individuals who had previously placed their trust in the healthcare system and vaccination initiatives. The outcome is an escalating count of individuals who hesitate or deliberately decline vaccination, resulting in decreased vaccination rates and heightened susceptibility to diseases that vaccines can prevent [50, 51]. Consequently, it is fundamental to acknowledge the apprehensions of individuals who are hesitant towards vaccines and furnish precise information to refute the misinformation propagated by the anti-vaccine movement.

### Vaccine hesitancy in low-, middle- and high-income countries

Vaccine hesitancy has been found to be associated with a range of socio-economic and demographic variables. The prevalence of concerns regarding the safety and effectiveness of vaccines is observed to be higher in high-income countries (HICs), as opposed to low- and middle-income countries (LMICs), where factors such as cultural and religious convictions, unfavorable past encounters with foreign medical interventions and vaccination initiatives, and challenges within healthcare systems are more prevalent [52]. Common factors between the two categories encompass a lack of trust in medical institutions and governmental bodies, the spreading of conspiracy theories, and the dissemination of misinformation through social media [19, 52].

Parents who declined to vaccinate their children or held a pessimistic outlook towards vaccination were found to be more susceptible to demonstrating such apprehensions [53]. The primary rationale cited by parents in India, Nigeria, and Pakistan for abstaining from vaccinating their children was the perceived risk of adverse effects associated with immunization. The apprehension regarding severe adverse effects may stem from prior encounters with unfavorable incidents after immunization, which may be attributed to the vaccination process [54–56]. This, together with the belief that vaccines may cause harm, has led to the perception that vaccinations result in adverse reactions such as fever. Furthermore, a commonly reported conjecture was that the polio vaccine administration was linked to adult sterility, leading to a significant number of parents declining to immunize their children with the vaccine [56].

In the Indonesian context, vaccine hesitancy can be attributed to various factors, given the country’s middle-income status. The complexity of the issue presents a significant challenge [26]. Vaccine hesitancy in Indonesia is a multifaceted problem that requires tailored and collaborative efforts across various sectors. Despite the government’s initiatives to improve vaccination rates, there remains a substantial gap in our understanding of the factors influencing vaccine acceptance and hesitancy [26, 57].

Furthermore, it is critical to highlight the significant disparities in vaccine coverage observed across Indonesia’s nationwide measles and rubella (MR) immunization program. Coverage rates vary widely among districts, ranging from as low as 2% to as high as 100%. Notably, more than one-third of districts report coverage rates below the established threshold of 70%. The link between the discontinuation of vaccination programs due to hesitancy and the subsequent decline in coverage rates is well-established [26].

Moreover, the hesitancy to receive the COVID-19 vaccine in Indonesia has been found to be highly correlated with various socio-demographic characteristics, including age, residential location, educational attainment, employment status, and family economic situation. Participants from Indonesia, Myanmar, Thailand, and Vietnam exhibited a higher degree of hesitancy towards receiving COVID-19 vaccines compared to their counterparts from the Philippines [58].

Additionally, concerns about vaccine safety have played a substantial role in shaping public discourse. Negative perceptions of vaccine safety, including anxieties about the rapid pace of vaccine development, have been identified as a primary driver of hesitancy. In low- and middle-income countries (LMICs) like Indonesia, where documented COVID-19 cases and fatalities have been relatively lower, individuals may perceive the disease as less severe, leading to reduced willingness to accept any potential risks associated with vaccination [59].

Finally, it is worth emphasizing that confidence in routine vaccinations has declined amid the ongoing COVID-19 pandemic. This trend has been observed in numerous countries, with some experiencing a significant decrease of up to 44 percentage points. The diminishing confidence level, coupled with the unique challenges faced by LMICs, has further exacerbated vaccine hesitancy in Indonesia [60].

In high-income countries, vaccine hesitancy could originate from complacency, as vaccine-preventable diseases have declined in these regions. In 2019, there were more than 1200 reported measles cases across 31 states in the United States [61]. This trend can be partially attributed to vaccine hesitancy [62]. Certain parents resisted vaccinating their children because of concerns regarding vaccines’ safety and probable negative consequences. Meanwhile, a few others declined vaccination due to their religious or philosophical convictions. The epidemic underscored the necessity for enhanced instruction and consciousness regarding the importance of immunizations, alongside endeavors to counteract the dissemination of false information concerning immunizations and enhance immunization availability. In Europe, there have been recent outbreaks of vaccine-preventable diseases such as measles and mumps [27, 63], which have been attributed to vaccine hesitancy. Vaccine hesitancy in certain nations is linked to a dearth of confidence in governmental and healthcare establishments, alongside a conviction that vaccines are superfluous owing to advancements in sanitation and hygiene. These outbreaks have led to demands for heightened vaccination rates and initiatives aimed at addressing vaccine hesitancy through public awareness drives and improved availability of vaccines.

Moreover, a contentious issue exists regarding the administration of the human papillomavirus (HPV) vaccine, which further exacerbates hesitancy [34]. Although the vaccine has demonstrated effectiveness in preventing cervical cancer and other diseases associated with HPV, some parents in developed countries are unwilling to immunize their children due to worries regarding the vaccine’s safety and potential negative consequences. The safety concerns surrounding the HPV vaccine were subject to investigation in Denmark [64]. The media initiated coverage of purported unfavorable occurrences concerning Danish females, encompassing a documentary that portrayed a cohort of girls exhibiting diverse incapacitating symptoms that were presumed to have been induced by HPV vaccination. The findings indicate a rapid decline in the utilization of HPV vaccination in the specified nation during the period spanning from 2009 to 2014 [64]. In certain instances, the reluctance has been intensified by inaccurate information propagated through social media and other communication platforms. As a result, the vaccination rates for HPV in certain high-income nations have persisted below the recommended levels set by public health authorities, leading to a continued susceptibility to HPV-associated illnesses among those who have not received the vaccine.

## Impact of vaccine hesitancy to the COVID-19 pandemic

The influence of vaccine hesitancy on the control and eradication of COVID-19 is significant and multifaceted. To begin with, slower progress in ending the pandemic because the slower people are to get vaccinated, the longer it will take to achieve herd immunity [65]. Next, vaccine hesitancy also increases the risk of people getting infected with COVID-19 and experiencing severe health complications, including hospitalization and death, particularly for vulnerable populations, such as the elderly and those with underlying health conditions [66]. The pandemic has already had a significant impact on the global economy, and vaccine hesitancy can amplify this effect [15]. Slower progress toward eradicating the pandemic may result in extended lockdowns and other restrictions [66].

Vaccine hesitancy can lead to unfilled appointments and vaccine doses being wasted. This concern is particularly true for mRNA vaccines such as Pfizer-BioNTech and Moderna, which have a shorter shelf life and require ultra-cold storage [67]. When people do not show up for their appointments, it can lead to vaccine doses being thrown away, which is a wasted resource. Hesitancy could also burden the healthcare system if large numbers of people remain unvaccinated, it can lead to increased healthcare costs due to ongoing hospitalizations and treatment for COVID-19 patients [68].

**Fig. 2:**
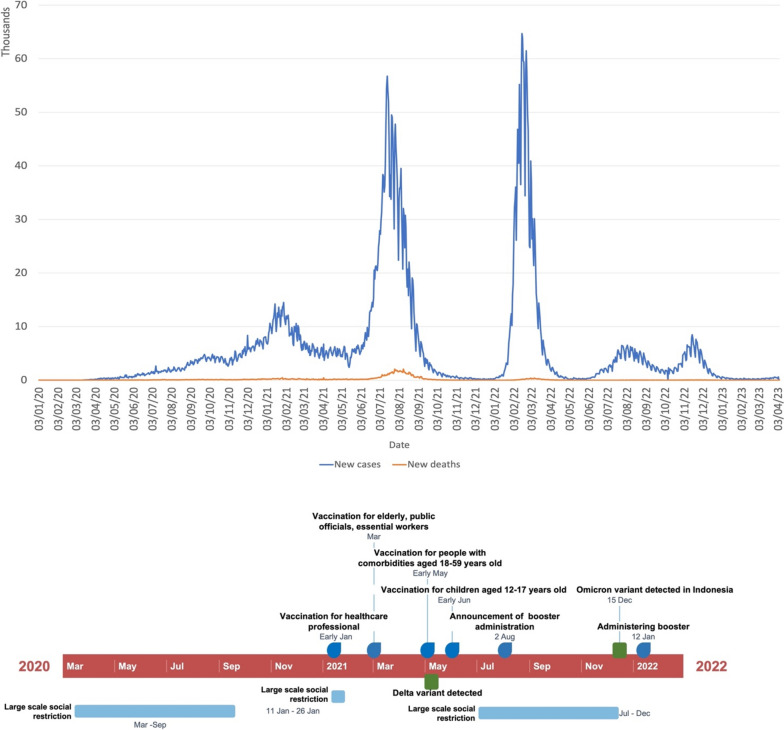
The number of new COVID-19 cases and the timeline of pharmaceutical and non-pharmaceutical interventions (NPIs) in Indonesia. The number of cases data was obtained from the WHO dataset, which is open to the public [69]

Indonesia has been facing a severe COVID-19 crisis since early 2020. Despite implementing various measures to control the spread of the virus, such as lockdowns and social restriction guidelines, the number of cases and deaths have remained high (Fig. 2). The highest number of cases was in June–September 2021 and January–May 2022. In early 2022, the number of Omicron variant infections was increasing. However, since the booster vaccination has been administered, the number of cases has decreased significantly.

The challenge of achieving the national vaccination target persists despite the widespread distribution of vaccines because of hesitancy. Before the pandemic, Indonesia experienced a significant degree of vaccine hesitancy, which led to suboptimal vaccination rates for illnesses such as measles and polio. The persistent distrust towards vaccines has had a lasting impact on the nation’s approach to addressing the epidemic caused by COVID-19. The introduction of COVID-19 vaccines in Indonesia was initially met with skepticism and reluctance among specific population segments because of apprehensions regarding the safety and efficacy of the vaccines.

**Fig. 3:**
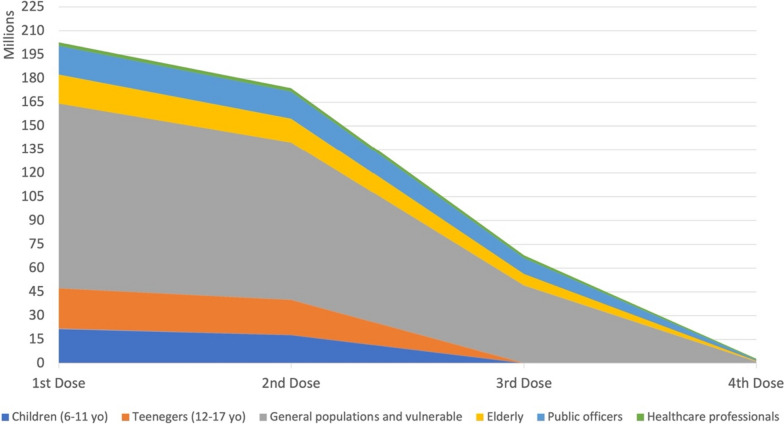
COVID-19 vaccination coverage based on the prioritization strategy in Indonesia (June 6th, 2023). Data were collected from Ministry of Health of Indonesia database [16]

Despite the government’s efforts to address vaccine hesitancy in Indonesia through the implementation of public awareness campaigns that utilize social media and influencers to emphasize the importance of vaccination, it remains an important barrier to the country’s efforts to control the spread of COVID-19. As of June 2023, the vaccination coverage for COVID-19 among the population remains relatively low. The rates for the first, second, third, and fourth doses are 86.87, 74.53, 37.93, and 1.76%, respectively, as depicted in Fig. 3. Based on the available data, Indonesia strives to achieve its national vaccination objective.

This low vaccination rate has resulted in many COVID-19 cases and deaths in Indonesia. It has also led to the emergence of new variants of the virus, which have caused further challenges in controlling the pandemic. Overall, vaccine hesitancy has significantly impacted the COVID-19 crisis in Indonesia, and efforts to address this issue remain crucial in the country’s ongoing fight against the virus.

## Challenges in reducing vaccine hesitancy

Preventing vaccine hesitancy, particularly concerning COVID-19 vaccines, presents many challenges. One of the major obstacles is the absence of confidence in the vaccine’s safety and effectiveness. This distrust could be due to apprehensions regarding the rapid production and authorization of the vaccine, lack of trust in governmental or healthcare institutions, or the dissemination of false information [70]. The amplification of misinformation by influential people or its alignment with pre-existing beliefs or concerns can pose a more significant challenge. Regarding safety, the documented adverse effects of COVID-19 vaccines may amplify vaccine hesitancy, particularly if individuals encounter unfavorable responses or harbor apprehensions regarding the vaccine’s enduring impacts [70, 71].

In addition, the acceptance of vaccines may be hindered by unequal access to them, which may be due to inadequate availability or dissemination in specific areas or demographics. The disproportionate distribution of vaccines in affluent nations and the limited accessibility in developing nations may exacerbate vaccine hesitancy and skepticism, particularly if specific demographics are perceived to be receiving higher priority.

### Vaccine inequity

The phenomena of vaccine hesitancy and vaccine inequity are interrelated and have the potential to mutually impact each other. Although they are different concepts, they can mutually strengthen one another and pose barriers in attaining significant vaccination coverage.

**Fig. 4:**
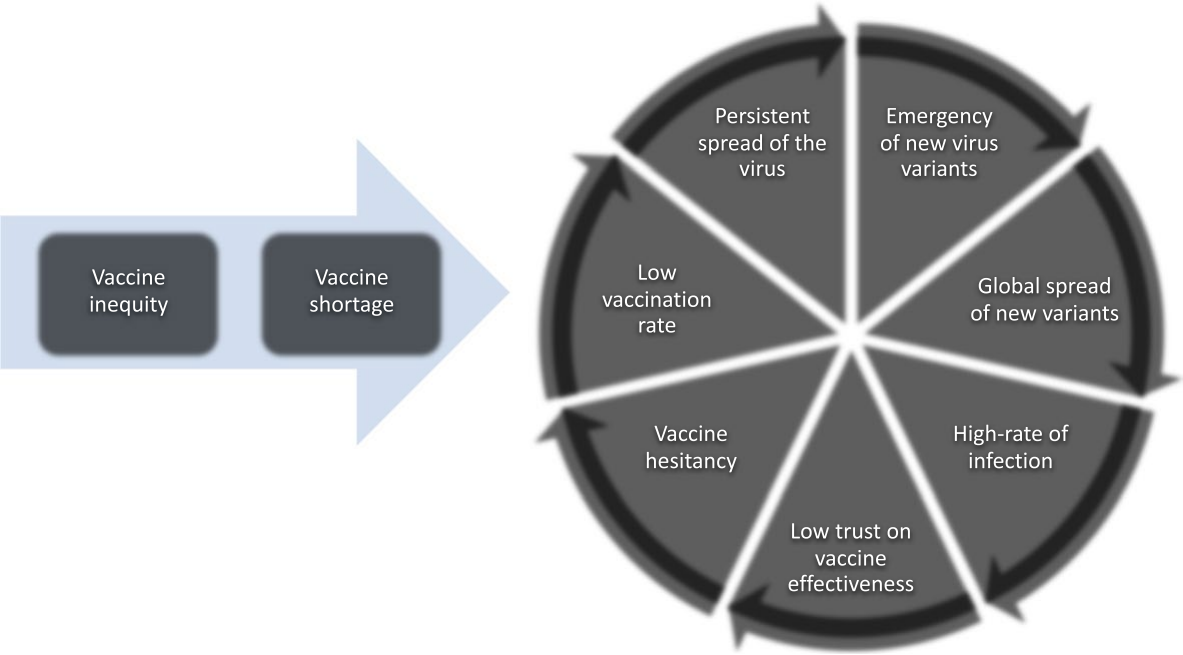
Association between vaccine inequity and vaccine hesitancy. Adapted from Gudina et al. [72]

Despite the current effectiveness of vaccination as a primary measure in mitigating the severity of COVID-19 infection, there is still a certain level of skepticism regarding its efficacy, largely due to the emergence of novel variants of the virus. A previous study referred to the association between vaccine inequity and vaccine hesitancy as a “vicious cycle” [72]. Vaccine inequities result in lower vaccination rates among certain populations, which can lead to the emergence and spread of new variants of concern (VOCs), such as the Omicron and Delta variants of COVID-19. These variants cause an increase in infection cases and mortality rates. The persistent spread of the virus globally can result in high infection rates, even among fully vaccinated individuals. This high infection rate can erode trust in the effectiveness of vaccines, further fueling hesitancy to get vaccinated. Ultimately, this vaccine hesitancy contributes to a decline in vaccination rates (Fig. 4).

Although vaccinated individuals have a reduced risk of experiencing severe COVID-19 symptoms, the emergence of breakthrough infections can create uncertainty within the population regarding the effectiveness of vaccines. This situation possesses the capacity to diminish the confidence of the general public and foster a broader sentiment of doubt towards immunization.

Amidst the COVID-19 pandemic, the issue of vaccine equity has emerged as a crucial concern, given the key function of vaccine accessibility in minimizing the transmission of the virus and limiting its detrimental impact on public health and the economy [73, 74]. Several factors, such as inadequate vaccine availability, insufficiencies in healthcare infrastructure, disparities in socioeconomic and geographic conditions, and vaccine nationalism, have delayed the achievement of vaccine equity. The concerns mentioned earlier have led to disparities in the distribution of vaccines, resulting in specific demographics being marginalized [74].

The task of achieving vaccination equity in Indonesia presents a significant challenge due to the prevalence of misinformation and limited understanding regarding vaccines, which can foster vaccine hesitancy and exacerbate disparities in vaccine distribution [26]. This challenge is particularly pertinent in rural regions, where access to accurate information may be limited. As previously mentioned, socioeconomic barriers, such as income and education levels, can contribute to vaccination inequities in Indonesia [52]. Research has shown a correlation between lower socioeconomic status, lower parental education levels, and a decreased likelihood of vaccination among children, further contributing to disparities in immunization coverage [30, 52].

Furthermore, Indonesia’s decentralized healthcare governance system, coupled with its vast geographical expanse, can hinder the distribution and accessibility of vaccines [75]. The transportation of vaccines from urban centers to geographically remote and isolated regions, often characterized by inadequate transportation networks, presents a significant obstacle. In many cases, reaching these remote areas requires the use of various modes of transportation. It is important to note that rural regions in Indonesia often face challenges in terms of infrastructure for vaccine storage and delivery. These limitations can lead to delays and pose a risk to the integrity of the cold chain, potentially compromising the quality of vaccines. Consequently, this situation can result in disparities in vaccination accessibility, with certain groups experiencing restricted access to vaccines due to logistical barriers.

### Vaccine nationalism

The concept of “vaccine nationalism” refers to a nation placing greater emphasis on fulfilling its own domestic vaccine requirements as opposed to those of other nations [76]. The act of accumulation of vaccines can lead to a constrained worldwide supply, thereby worsening the pre-existing disparities in vaccine accessibility between countries with high income and those with low-middle income [76, 77]. The phenomenon of vaccine nationalism has emerged as a significant issue amidst the COVID-19 pandemic. Several affluent nations have procured substantial quantities of vaccines for their respective populations [76], thereby restricting the availability of vaccines for countries with lower-middle income. The outcome of this situation could hinder the timely distribution of vaccines, leading to limited accessibility of vaccines for marginalized communities residing in low to middle-income nations.

A potential correlation could be present between vaccine hesitancy and vaccine nationalism. Within the framework of vaccine nationalism, which refers to a country’s prioritization of its vaccine requirements over those of other nations, specific individuals may believe that vaccines being disseminated are of inferior quality or less secure, mainly if they are produced in foreign countries. This phenomenon could exacerbate vaccine hesitancy and engender a disinclination to undergo vaccination.

In the context of the COVID-19 pandemic in Indonesia, vaccine nationalism has had a significant impact on the country’s vaccination efforts and overall response to the crisis. Vaccine nationalism has led to a limited supply of vaccines for low- and middle-income countries like Indonesia, as high-income countries secure a significant portion of the global vaccine production capacity. This limited availability has hindered the country’s vaccination rollout and contributed to vaccine hesitancy among the population.

Moreover, by June 2021, Indonesia had only secured around 80 million doses of Sinovac and AstraZeneca, covering 40 million of the most at-risk citizens, which accounted for only 22% of the total population eligible for vaccination [78]. Therefore, the perception of unfair distribution caused by vaccine nationalism has further eroded trust in vaccines and increased hesitancy in Indonesia. As wealthier countries receive a larger share of vaccines, people in Indonesia may question the effectiveness and fairness of the vaccination efforts. Despite President Joko Widodo’s criticism of affluent nations for obstructing the global vaccine supply due to vaccine nationalism, Indonesia still struggles to secure an adequate number of vaccine doses to immunize its population and control the spread of COVID-19 [79, 80].

Therefore, it is important to prioritize to worldwide collaboration and fair allocation of vaccines. The endeavors above encompass augmenting vaccine production, enhancing vaccine distribution networks, and guaranteeing equitable and impartial distribution of vaccines to all demographics. For instance, the COVAX program is a global initiative that promotes vaccine equity by providing equal access to vaccines for all nations [81]. This program can significantly reduce vaccine nationalism and enhance vaccine acceptance and uptake rates, thereby contributing to achieving global vaccination coverage.

## Strategies reducing vaccine hesitancy in Indonesia

The administration of vaccines is presently crucial in reducing the transmission of COVID-19 and achieving herd immunity. The global distribution and acceptance of vaccines have exhibited disparities, particularly in regions with low- and middle-income countries [82]. These disparities are attributed to insufficient knowledge, logistical challenges, inadequate equipment, and financial constraints related to vaccination [82, 83]. Moreover, the appearance of novel virus strains has presented supplementary obstacles to managing the pandemic.

Achieving herd immunity via vaccination is a multifaceted and continuous endeavor. The attainment of herd immunity for COVID-19 necessitates a substantial portion of the populace to possess immunity to the virus. To illustrate, the Indonesian authorities aimed to immunize at least 70% of the population to accomplish herd immunity [84]. The prioritization strategies for COVID-19 vaccination entail identifying groups that should be given priority access to the vaccine, considering factors such as vaccine availability, public health directives, and the prevailing epidemiological conditions in the locality. Various approaches are commonly employed to prioritize individuals for vaccination. These include prioritization based on age, occupation, health status, geographic location, equity considerations, and risk of exposure.

Indonesia experienced several challenges in vaccinating its population against COVID-19, including limited vaccine supply and logistical barriers in reaching remote and conflict-affected areas. Several strategies have been evaluated to improve the effectiveness of immunization. Initially, the government prioritized the immunization of vulnerable populations, including healthcare professionals, senior citizens, and those with underlying medical conditions.

Subsequently, the government expanded the number of vaccination sites and services, introducing mobile vaccination units, pop-up clinics, and drive-through centers. These initiatives aimed to increase vaccine accessibility for individuals residing in geographically isolated regions, addressing the challenges they faced in reaching vaccination facilities. In addition, a robust tracking system was implemented to monitor vaccine coverage and identify gaps in the vaccination campaign, enabling targeted interventions and program adjustments to improve its efficiency.

Moreover, public health campaigns were launched to boost vaccine acceptance and reduce hesitancy. To combat hesitancy arising from concerns about inequity and vaccine availability, the government provided COVID-19 vaccines free of charge and communicated this clearly to the public. This step eliminated financial barriers and boosted vaccine uptake.

To bolster vaccine availability, the government secured agreements with multiple vaccine manufacturers, emphasizing domestic vaccine production and engaging in vaccine diplomacy. This diplomatic effort resulted in Indonesia receiving support in the form of over 40 million doses of COVID-19 vaccines from the United States [85]. Additionally, collaborating with international partners, such as UNICEF, further ensured fair and equitable access to COVID-19 vaccines, guaranteeing a sufficient vaccine supply and supporting distribution efforts [86].

Furthermore, the World Bank has extended support to Indonesia’s pandemic response and vaccination program, with the goal of providing free COVID-19 vaccines to its adult population of 181.5 million people [87]. The financial assistance offered by the World Bank will contribute to enhancing the resilience of Indonesia’s healthcare system and reinforcing its ability to conduct surveillance, including testing and tracing of new COVID-19 cases and genomic surveillance to monitor emerging variants [88].

Addressing concerns about non-halal vaccine ingredients, the government involved the Indonesian Ulama Council (MUI), the country’s highest Muslim clerical council, in the COVID-19 vaccine procurement and certification process. This collaboration reassured religious communities about the safety and halal status of the vaccines. Furthermore, the Indonesian government established partnerships with various countries and organizations to procure COVID-19 vaccines, ensuring access to a diverse range of vaccines, including those that meet halal requirements and are suitable for the Indonesian population. Additionally, clinical trials for COVID-19 vaccines, including those from Sinovac Biotech, were facilitated, with Indonesian clerics inspecting the production facilities and processes to provide assurance of their safety and halal status.

To further promote vaccination equity and increase uptake, the Indonesian government has introduced a phased vaccination program that prioritizes healthcare workers, the elderly, the general and at-risk population, and children [89]. Additionally, the implementation of online vaccination registration has been demonstrated to enhance the public’s willingness to receive vaccination due to the convenience it provides in accessing vaccines, and the integrated technology utilized to store vaccination data, which is accessible by both individuals and the government. The system was formerly known as PeduliLindungi but is now called SATUSEHAT and is managed by the Ministry of Health of Indonesia.

In order to mitigate vaccine hesitancy and safeguard public health, healthcare professionals, public health authorities, and other relevant parties must collaborate to furnish precise information regarding vaccines and to counteract apprehensions and misconceptions surrounding vaccine safety. The process may entail fostering community engagement to establish confidence and tackling the fundamental causes of vaccine hesitancy, such as skepticism towards the healthcare system. Table 2 outlines various strategies that can be employed to enhance vaccination rates.

**Table 2:** Approaches to decrease hesitancy and enhance vaccine uptake

Type of Strategy	Description
Vaccine availability and equitability	Expanding the distribution of vaccines, particularly in hard-to-reach regions, to guarantee equitable rights and accessibility for all citizens.
Support from the military (if necessary)	Engaging the national military in vaccination efforts to promote vaccination uptake and enhance security during the vaccination process in regions currently experiencing conflict.
Public health campaign	Raising public consciousness regarding the significance of vaccination and its long-term beneficial outcomes. Engaging community leaders, including those from religious and community organizations, in public health campaigns is a potential strategy to foster confidence and promote vaccine uptake within the community.
Addressing negative stereotypes	Conducting extensive counseling sessions to disseminate accurate information about vaccines, potential side effects, and appropriate actions to take in the event of experiencing side effects.
Counseling from healthcare workers	While the internet is the primary source of vaccine information for the general public, healthcare workers are regarded as the most reliable source. They can offer guidance to patients or guardians on vaccination and provide transparent details on potential side effects in order to foster public confidence in the use of vaccines.

The World Health Organization (WHO) has taken several steps to improve the COVID-19 vaccination campaign globally, including providing guidance, monitoring the safety of COVID-19 vaccines, training healthcare workers, and helping to establish vaccination sites [90]. The Indonesian government has adopted various approaches to enhance vaccination, including collaborating with the national military. Although vaccination cannot be imposed, partnering with the national military can promote vaccination significance and expand coverage to certain facilities such as schools, workplaces, and conflict-affected regions. While it may be necessary to engage the military in vaccination efforts in conflict-affected areas, caution should be taken as some studies have shown that military involvement can decrease trust in the government and raise suspicions about hidden agendas behind the vaccination program.

The challenges related to vaccine access and distribution in conflict-affected areas are further exacerbated by political, logistical, security, and resource-related complications. These challenges are part of the broader global issues surrounding COVID-19 vaccine development, production, procurement, and distribution, which affect populations worldwide. Despite an increase in vaccine production, a significant portion of the limited supply has been secured by high-income countries through nontransparent commercial contracts, while COVAX has faced funding shortfalls. Conflict-affected areas face even more significant hurdles due to resource scarcity, logistical difficulties, competing priorities, and insecurity.

To address these challenges, vaccination programs should leverage existing humanitarian logistical capacity, include conflict-affected areas in national deployment and vaccination plans, and distribute vaccines at transit points for migrants or camps for refugees or internally displaced people. Vaccinators should be protected from potential threats and humanitarian access may need to be negotiated. Vaccination programs must follow impartiality principles to avoid the perception of favoritism, and community engagement should be prioritized rather than partnering with military or security personnel.

## Reflection and further recommendations

The issue of vaccine hesitancy has posed a considerable obstacle in managing the COVID-19 outbreak, particularly in nations with limited economic resources such as Indonesia. Elevated levels of vaccine hesitancy may result in decreased vaccination rates and hindered advancement toward mitigating the dissemination of the virus. In order to tackle this matter, it is imperative to implement public health campaigns and community engagement initiatives to promote vaccine acceptance and uptake.

Enhancing public awareness regarding the advantages of vaccination and mitigating prevalent apprehensions and misunderstandings concerning vaccine safety and effectiveness is a crucial short-term strategy. This objective can be attained by implementing focused communication initiatives that leverage diverse media platforms, including social media, radio, and television. It is recommended that governments engage reputable community leaders and influencers in their vaccination initiatives, as they can effectively debunk misconceptions and promote vaccine acceptance. One feasible strategy in the short run is to guarantee the availability of vaccines to every qualified person. The aforementioned entails the resolution of hindrances to entry, such as linguistic and transportation obstacles, and guaranteeing equitable allocation of vaccines among diverse regions and demographic categories. Governments must prioritize developing and enhancing their healthcare systems, which encompasses establishing and reinforcing vaccination infrastructure and workforce in the context of long-term planning. This point would facilitate their ability to efficiently administer vaccinations to their respective populations and effectively address potential future pandemics.

The strategies above have demonstrated a degree of efficacy in augmenting vaccination rates. However, continued endeavors are essential to enhance vaccine uptake. In Indonesia, various measures have been employed to enhance vaccination coverage, such as according to priority to immunization for susceptible groups, dispatching mobile vaccination teams to geographically isolated regions, and initiating public health advocacy programs to augment vaccine uptake.

Enhancing vaccine uptake and reducing vaccine hesitancy in rural areas may require special strategies, as factors such as demographics, strong cultural influences, and limited knowledge about vaccines often play a major role in hesitancy. Therefore, it is significant to identify and involve local leaders, community organizations, and trusted individuals in combating vaccine misinformation and addressing questions from their peers, thus engaging trusted voices within the community. In addition to this, collaborating with local community-based organizations, including religious institutions, schools, and non-profit groups, to organize vaccination events, provide education, and address specific concerns related to COVID-19 vaccines can be highly effective. Such partnerships enable reaching underserved populations and building trust in the vaccination process.

Furthermore, developing targeted communication materials and strategies that are culturally appropriate and accessible to rural communities can improve knowledge and awareness. This might involve using local languages, visual aids, and storytelling techniques to effectively convey information about COVID-19 vaccines and address common concerns.

Additionally, addressing health equity issues, such as logistical challenges in rural area, can significantly reduce hesitancy. Ensuring that COVID-19 vaccination sites are easily accessible to rural residents can be achieved through the establishment of mobile vaccination units, pop-up clinics, and partnerships with local healthcare providers. This approach helps overcome transportation and distance-related barriers, making it more convenient for rural residents to access vaccines.

As a result of some of the mentioned programs, UNICEF data indicates that Indonesia has achieved a 94.6% full childhood immunization coverage, surpassing the national target of 94.1% [91]. This signifies an improvement in childhood immunization coverage within the country. However, regarding COVID-19 vaccination, the current vaccination rate in Indonesia is unclear. Nevertheless, search results suggest that the rate has been showing signs of improvement over time.

Continuous monitoring and evaluation of how hesitancy influences vaccine uptake is essential. While Indonesia has attained high coverage for the initial two doses of COVID-19 vaccines, there has been a decline in the administration of booster shots. The decreasing interest in vaccination among the general population, combined with a reduction in the number of COVID-19 cases, has further hindered efforts to increase booster dose coverage. This situation underscores the necessity of implementing a catch-up immunization campaign, which can be adapted to local culture to reach a larger segment of the population effectively.

The experience of Indonesia with vaccine hesitancy and vaccination strategies could offer valuable insights for other nations encountering comparable obstacles. Efforts to prioritize vulnerable populations and enhance vaccine accessibility in remote areas may prove to be particularly efficacious in nations with substantial rural demographics. Public health campaigns and community engagement initiatives aimed at dispelling prevalent misconceptions regarding vaccines have the potential to foster trust and enhance vaccine acceptance.

It is necessary to acknowledge that vaccine hesitancy is a multifaceted matter that is impacted by various factors, such as cultural and religious convictions, skepticism towards healthcare systems, and dissemination of inaccurate information. The development of practical approaches to tackle vaccine hesitancy will require customization to suit particular cultural and societal settings, and continuous assessment and appraisal will be necessary to gauge the efficacy of such interventions.

In conjunction with the strategies mentioned previously, an alternative methodology that may prove efficacious in preventing vaccine hesitancy is using social networks and peer influence. Research has indicated that social networks and peers tend to have a more significant impact on individuals than public health campaigns or authoritative figures. Thus, endeavors to involve and activate community leaders, influencers, and peer networks may prove efficacious in augmenting vaccine acceptance and uptake [60].

Moreover, it is important to acknowledge the impact of vaccine nationalism on the exacerbation of vaccine inequity and hesitancy. Nations with abundant resources and superior vaccination rates must prioritize the equitable distribution of vaccines globally. Addressing concerns about unequal access to vaccines and promoting the notion that every individual has a right to protection against the virus can mitigate vaccine hesitancy.

Finally, it is essential to acknowledge that mitigating vaccine hesitancy is not solely significant within the confines of the COVID-19 pandemic but also for fostering and preserving confidence in the healthcare infrastructure. Mitigating vaccine hesitancy can foster confidence in healthcare systems and enhance the probability of individuals pursuing and obtaining other vital preventive healthcare services.

The Indonesian government has implemented measures to attain the national objective of attaining herd immunity through the sustained administration of COVID-19 vaccinations. Furthermore, the government has implemented independent manufacturing of the COVID-19 vaccine to enhance the accessibility of the domestic vaccine. Continued endeavors to extend vaccination services to individuals residing in remote regions are underway, and health measures are being enforced to mitigate the incidence of new infections.

Although new strains of the COVID-19 virus have surfaced, the administration of vaccines has decreased the intensity of symptoms in contrast to the initial outbreak of the virus. It is anticipated that in the future, COVID-19 will assume a resemblance to the common cold and cease to present a substantial menace. It is plausible that COVID-19 may eventually be eliminated, similar to polio eradication. Therefore, including of the COVID-19 vaccine in the national vaccination program is imperative to ensure consistent vaccination for the population of Indonesia. Anticipated is a reduction in vaccine hesitancy, not solely for the COVID-19 vaccine but also other government-mandated national vaccination initiatives. Further research is required to evaluate the most effective COVID-19 vaccine in addition to the National Immunization Program. This evaluation should consider cost and clinical efficacy concerning dosage, population characteristics, and comorbidities.

Moreover, it is imperative to acknowledge that the issue of vaccine hesitancy is not novel and is not restricted to low- and middle-income nations. The matter at hand is intricate and subject to the influence of various factors, such as past events, cultural convictions, and socioeconomic standing. Hence, the resolution of vaccine hesitancy necessitates a thorough and culturally attuned strategy that considers the specificities and circumstances of the given locality.

## Conclusion

The hesitancy to receive vaccines has been recognized as a noteworthy obstacle in the worldwide endeavors to manage the COVID-19 outbreak. Vaccine hesitancy is a multifaceted phenomenon that can be shaped by various determinants, such as skepticism towards healthcare institutions, cultural and religious convictions, and the spread of inaccurate information. Elevated levels of reluctance towards vaccination can lead to decreased vaccination coverage and exacerbate the continued transmission of the virus. Ensuring fair distribution of vaccines is imperative to guarantee universal accessibility to vaccination, irrespective of geographical location or financial status. Acknowledging the multifaceted nature of vaccine hesitancy, a comprehensive approach is necessary, encompassing public health initiatives, community involvement, and policy interventions. The promotion of vaccine acceptance and uptake among all populations is essential in the worldwide battle against COVID-19, and it is compulsory to address vaccine hesitancy in order to achieve this goal.

## Data Availability

The data utilized in this study is sourced from the provided references.
